# Residue Dynamics and Risk Assessment of Prochloraz and Its Metabolite 2,4,6-Trichlorophenol in Apple

**DOI:** 10.3390/molecules22101780

**Published:** 2017-10-20

**Authors:** Qingkui Fang, Gengyou Yao, Yanhong Shi, Chenchun Ding, Yi Wang, Xiangwei Wu, Rimao Hua, Haiqun Cao

**Affiliations:** 1 School of Plant Protection, Provincial Key Laboratory for Agri-Food Safety, Anhui Agricultural University, Hefei 230036, China; qkfang@163.com (Q.F.); chenchundingdcc@163.com (C.D.); 2 School of Resource & Environment, Provincial Key Laboratory for Agri-Food Safety, Anhui Agricultural University, Hefei 230036, China; yaogengyou@126.com (G.Y.); shiyh@ahau.edu.cn (Y.S.); wangyi@ahau.edu.cn (Y.W.); wxw@ahau.edu.cn (X.W.); rimaohua@ahau.edu.cn (R.H.)

**Keywords:** apple, prochloraz, 2,4,6-trichlorophenol, residue dynamics, risk assessment

## Abstract

The residue dynamics and risk assessment of prochloraz and its metabolite 2,4,6-trichlorophenol (2,4,6-TCP) in apple under different treatment concentrations were investigated using a GC-ECD method. The derivatization percent of prochloraz to 2,4,6-TCP was stable and complete. The recoveries of prochloraz and 2,4,6-TCP were 82.9–114.4%, and the coefficients of variation (CV) were 0.7–8.6% for the whole fruit, apple pulp, and apple peel samples. Under the application of 2 °C 2.0 g/L, 2 °C 1.0 g/L, 20 °C 2.0 g/L, and 20 °C 1.0 g/L treatment, the half-life for the degradation of prochloraz was 57.8–86.6 d in the whole fruit and apple peel, and the prochloraz concentration in the apple pulp increased gradually until a peak (0.72 mg·kg^−1^) was reached. The concentration of 2,4,6-TCP was below 0.1 mg·kg^−1^ in four treatment conditions and not detected (<LOD) in apple pulp. Finally, based on the detection of market samples in Hefei (China), we believe that the residual level of prochloraz in apples meets the requirements of the Chinese standards.

## 1. Introduction

Apples represent an important part of fruit production and agricultural trade, especially in China [1]. Apples are widely consumed because they are rich in phytochemicals, such as phenolics, flavonoids, and carotenoids, which are important for human health [2,3,4]. Epidemiological studies indicated that apples have been associated with a decreased risk of chronic diseases such as cardiovascular disease, cancer, and asthma [5,6]. Apples are prone to attack by several pests and diseases; in order to ensure the quality of apples, postharvest treatment of fungicides is essential [7].

Prochloraz [*N*-propyl-*N*-[2-(2,4,6-trichlorophenoxy)-ethyl]imidazole-1-carboxamide] is a broad-spectrum imidazole fungicide with certain contact poisoning and systemic action [8]. It was reported that prochloraz is widely used on fruit and vegetables to prevent the fruit from deterioration during storage [9,10,11]. The final metabolite of prochloraz is proven to be 2,4,6-trichlorophenol (2,4,6-TCP) in many plants. 2,4,6-TCP is characterized by significant toxicological effects and potential carcinogenicity, being listed as a priority pollutant by the US Environmental Protection Agency (EPA) [12]. Prochloraz and its metabolites such as 2,4,6-TCP are harmful to health [13].

There have been several studies on the residue behavior and dietary risk assessment of prochloraz in agroducts and environmental samples, such as determination of prochloraz residue in fruits [14], fruit juices [15,16], green tea soft drink [17], and cultivated mushrooms [18]. Additionally, studies have been conducted on the residual detection and degradation dynamics of prochloraz in Ya pears [19]; degradation dynamics and safety evaluation of prochloraz in bananas [20,21]; residue determination and dynamic research of prochloraz and its metabolite in oranges [22,23,24]; and residual dynamics and safety evaluation of prochloraz in ginseng and soil [25]. Scholars have paid more and more attention to prochloraz residue, which has provided us with valuable literature. However, there is a lack of studies on the dynamics of prochloraz and its metabolite 2,4,6-TCP during the storage of apples.

The aims of this study were (1) to establish a reliable and effective method for the detection of prochloraz and its metabolite 2,4,6-TCP in apples using a GC-ECD method, (2) to evaluate the residue dynamics and risk assessment of prochloraz and its metabolite 2,4,6-TCP in apples under different treatment concentrations, and (3) to determine the levels of prochloraz residues in apple samples collected from the Hefei, China.

## 2. Results and Discussion

### 2.1. Validation of Derivatization Percent

In this experiment, 10 mL of 0.1, 0.2, 0.8, and 1.0 mg/kg prochloraz working standard solution was added into the blank samples. The samples were tested and compared with the corresponding working standard solution of 2,4,6-TCP. The results showed that the derivatization percent of prochloraz to 2,4,6-TCP was stable and complete (Table 1). The average derivatization percent was 101.0%, and relative standard deviations (RSDs) were 4.2%. Thus, the derivatization method can be used for prochloraz determination.

### 2.2. Recovery Study

The limits of detection (LOD) of prochloraz and 2,4,6-TCP were 0.2 and 0.02 mg·kg^−1^, where a signal-to-noise ratio of at least 3 could be gained. Furthermore, the limit of quantification (LOQ) of 2,4,6-TCP was 0.05 mg·kg^−1^, confirmed by a spiked study at this level of 2,4,6-TCP. The analyte recoveries and precision values obtained from the validation study are summarized in Table 2. Prochloraz was added to the untreated control samples at 0.2, 1.0, and 2.0 mg·kg^−1^ for the whole fruit and apple pulp and 0.25, 1.0, and 5.0 mg·kg^−1^ for the apple peel. 2,4,6-TCP was added to the untreated control samples at 0.02, 0.2, and 2.0 mg·kg^−1^ for the whole fruit and apple pulp. The mean values of three replicates at each spiked level were recorded. The recoveries of prochloraz ranged from 82.9 to 114.4%, 2,4,6-TCP ranged from 84.7 to 109.1%, and the coefficients of variation (CV) were 0.7–8.6% for the 15 samples. Thus, these results demonstrated that the GC method can be used for apple sample determination.

### 2.3. Residue Dynamics of Prochloraz in Apple

**Figure 1:**
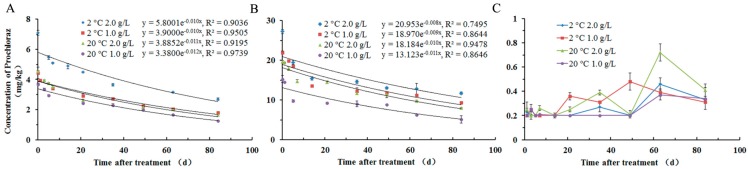
Residual dynamics of Prochloraz in apple under different treatment concentrations (*n* = 3). (**A**) Whole fruit; (**B**) apple peel; (**C**) apple pulp.

The data on residue dynamics obtained for prochloraz in apples are shown in Figure 1. A gradual and continuous deterioration of prochloraz residues in the whole fruit (Figure 1A) and apple peel (Figure 1B) samples was observed. The average residues of prochloraz in whole fruit (Figure 1A) were 7.1, 4.4, 4.6, and 3.7 mg·kg^−1^ respectively, after 2 h of the application of 2 °C 2.0 g/L, 2 °C 1.0 g/L, 20 °C 2.0 g/L, and 20 °C 1.0 g/L treatment. The half-life (T_1/2_) for the degradation of prochloraz in the whole fruit was calculated to be 69.3 days, 69.3 days, 63.0 days, and 57.8 days (Table 3), respectively. Furthermore, the average residues of prochloraz in apple peel (Figure 1B) were 27.3, 21.9, 19.7, and 15.3 mg·kg^−1^ respectively, after 2 h of the application of 2 °C 2.0 g/L, 2 °C 1.0 g/L, 20 °C 2.0 g/L, and 20 °C 1.0 g/L treatment. At the same time, the T_1/2_ for the degradation of prochloraz in the apple peel was calculated to be 86.6 days, 77.0 days, 69.3 days, and 63.0 days (Table 3), respectively.

The residue dynamics of prochloraz in the apple pulp are shown in Figure 1C. The prochloraz concentration in the apple pulp increased gradually until a peak was reached. These maximum concentrations of prochloraz in 2 °C 2.0 g/L, 2 °C 1.0 g/L, 20 °C 2.0 g/L, and 20 °C 1.0 g/L treatment were 0.46 mg·kg^−1^, 0.48 mg·kg^−1^, 0.72 mg·kg^−1^, and 0.37 mg·kg^−1^, respectively. Therefore, compared to the concentration of prochloraz in apple peel, the results showed that the transfer of prochloraz to the apple pulp is limited.

### 2.4. Residue Dynamics of 2,4,6-TCP in Apple

**Figure 2:**
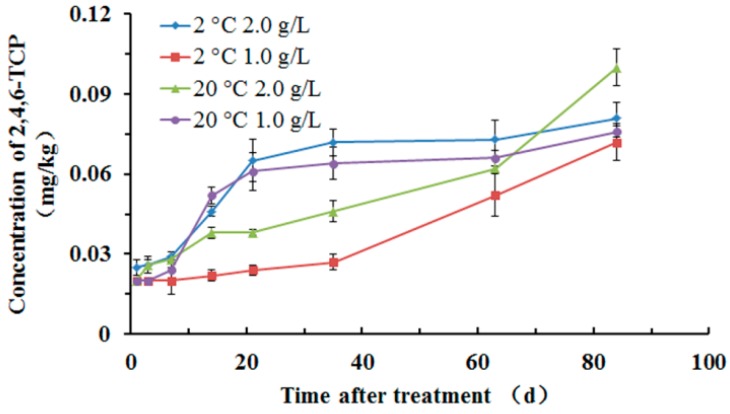
Residual behavior of 2,4,6-TCP in apple whole fruit.

A gradual and continuous increase of 2,4,6-TCP residues in the whole fruit samples was observed (Figure 2). Because 2,4,6-TCP is the final metabolite of prochloraz in many plants, the amount of 2,4,6-TCP increased gradually with the degradation of prochloraz. The results showed that the concentration of 2,4,6-TCP was below 0.1 mg·kg^−1^ in 2 °C 2.0 g/L, 2 °C 1.0 g/L, 20 °C 2.0 g/L, and 20 °C 1.0 g/L treatment conditions. Furthermore, 2,4,6-TCP was not detected (<LOD) in apple pulp. Additionally, according to Figure 2, the concentration of 2,4,6-TCP increased, probably because of the high treatment concentration of prochloraz, and the peak value of 2,4,6-TCP did not appear in 84 days.

### 2.5. Determination of Prochloraz Residue of Apple Samples in Hefei Market

Authentic apple samples were collected from fruit wholesale markets and supermarkets in Hefei, China. The detected results are shown in Table 4. In the 20 apple samples, the residual concentration ranged from below the LOD to 0.86 mg·kg^−1^, and the detection rate of prochloraz was 35%, which did not exceed the maximum residue limit (MRL) of prochloraz established by Chinese regulation in apples [26].

## 3. Materials and Methods

### 3.1. Chemicals and Instruments

Prochloraz (98.0%) and 2,4,6-TCP (98.5%) were purchased by Dr. Ehrenstorfer GmbH. The commercial formulations of 25% prochloraz emulsifiable concentrate (EC) were provided by Guoguang Agrochemical Ltd. (Jianyang, Sichuan, China). Acetone, hexane, petroleum ether, dichloromethane, and anhydrous sodium sulfate of analytical grade were purchased from Xilong Chemical Co., Ltd. (Shanghai, China).

An EYELA N-1100 rotary evaporator was used from the Shanghai Ailang Instruments Co., Ltd. (Shanghai, China). An SC-3610 low-speed centrifuge was used from Anhui Zhongke Zhongjia Scientific Instruments Inc. (Hefei, Anhui, China). A KQ-5200 ultrasonic cleaner was used from the Kunshan Ultrasonic Instrument Co., Ltd. (Shanghai, China). An SQ-2119B multifunctional food processing instrument was used from the Shanghai Shuaijia Electronic Technology Co., Ltd. (Shanghai, China). A DHG-9070A electrothermal oven thermostat blast was used from the Shanghai Yiheng Science and Technology Co., Ltd. (Shanghai, China). An SY-2 digital display room temperature sand bath was used from the Jintan Chenghui Instrument Factory (Jiangsu, China).

### 3.2. Postharvest Treatment and Storage

Prochloraz aqueous suspension was prepared by diluting the commercial formulation of 25% prochloraz emulsifiable concentrate (EC) with water. The treatments were carried out by dipping the apple samples (Red Fuji apple was purchased from Dangshan (Suzhou, China)) in prochloraz aqueous suspension for 2 min, and the treated samples were then dried in a ventilated area. Subsequently, the treated samples were stored in cartons at 20 °C and 2 °C, respectively. The dosages for the residue dynamics experiment were 1.0 and 2.0 g a.i./L (active ingredient per liter). Residue samples collected at 0 (2 h after treatment), 1, 3, 5, 7, 14, 21, 35, 49, 63, and 84 days after application were for the residue dynamics study.

### 3.3. Sample Extraction, Clean up, and Gas Chromatograpy (GC) Analysis

#### 3.3.1. Sample Preparation

The apple samples, including the apple peel and the whole fruit, were collected in postharvest treatment and the storage experiment. Pericarp thickness by mechanical means is 2 mm. The apple peel and the whole fruit were triturated separately using a household blender and homogenized for analysis.

#### 3.3.2. Extraction and Derivatization Process for Prochloraz Determination

The whole fruit (5.0 g) or apple peel (2.0 g) were extracted with 30 mL of acetone by thoroughly shaking for 30 min, centrifuging at 3500× *g* for 5 min, and transferring the supernatant to a 150 mL flask. An additional 30 mL of acetone was added to the samples, and the mixture was thoroughly shaken for 30 min and centrifuged at 3500× *g* for 5 min. The supernatant was transferred to the flask, and concentrated close to dryness at 40 °C. The residue was dissolved in 20 mL of water, and the flask was then washed with 20 mL of saturated salt water and 30 mL of CH_2_Cl_2_, respectively. The solution was transferred to a 250 mL separator funnel and thoroughly shaken for 5 min. The lower organic phase was transferred to a 150 mL flask. An additional 30 mL of CH_2_Cl_2_ was added to the 250 mL separator funnel and thoroughly shaken for 5 min. The lower organic phase was transferred to the 150 mL flask, dried over anhydrous sodium sulfate, and concentrated close to dryness at 40 °C. The obtained residues were prepared for the derivatization process.

Five grams of dry pyridine hydrochloride was added to the residues, and the flask was heated to 270 °C for 90 min in a sand bath. After cooling to room temperature, 20 mL of water was added to the flask, the flask was then washed with 30 mL of petroleum ether, furthermore the water and petroleum ether of the mixed solution were transferred to a 150 mL separator funnel and thoroughly shaken for 5 min. The organic phase was transferred to a 150 mL flask. An additional 30 mL of petroleum ether was added to the separator funnel and thoroughly shaken for 5 min. The organic phase was transferred to the flask, dried over anhydrous sodium sulfate, and concentrated close to dryness at 40 °C. The volume was set to 10 mL and placed in a volumetric flask with *n*-hexane to await measurement.

#### 3.3.3. Sample Treatment for 2,4,6-TCP Determination

The whole fruit samples (5.0 g) and apple pulp (5.0 g) were extracted with 30 mL of acetone by thoroughly shaking for 30 min, centrifuging at 3500× *g* for 5 min, and transferring the supernatant to a 150 mL separatory funnel. An additional 30 mL of acetone was added to the samples and the mixture was thoroughly shaken for 30 min and centrifuged at 3500× *g* for 5 min. The supernatant was transferred to the separatory funnel and concentrated close to dryness at 40 °C. The residue was dissolved in 20 mL of water, and the flask was then washed by 20 mL of saturated salt water and 30 mL of CH_2_Cl_2_, respectively. The solution was transferred to a 250 mL separator funnel, and thoroughly shaken for 5 min. The lower organic phase was transferred to a 150 mL flask. An additional 30 mL of CH_2_Cl_2_ was added to the 250 mL separator funnel, and the contents were thoroughly shaken for 5 min. The lower organic phase was transferred to the 150 mL flask, dried over anhydrous sodium sulfate, and concentrated close to dryness at 40 °C.

A Florisil SPE was activated with 5 mL of acetone, and then with 5 mL *n*-hexane. The extract was washed with *n*-hexane and transferred to the SPE column, which was rinsed by an acetone:*n*-hexane (*v*/*v* = 1:1) mixed solution to remove interfering substances. The eluent was discarded. The SPE column was then eluted with an acetone:*n*-hexane (*v*/*v* = 9:1) mixed solution, and the eluate was concentrated to dryness at 40 °C. The volume was set to 10 mL and placed in a volumetric flask with *n*-hexane to await measurement.

#### 3.3.4. GC Condition

GC analysis was performed on a GC-2010plus system (Shimadzu, Japan) coupled with an electron capture detector (ECD). A 30 m × 0.25 mm × 0.25 μm HP-5 capillary column (Agilent Technologies, Santa Clara, CA, USA) was used. The injector was held at 240 °C and operated in splitless mode (volume injected 1 μL). The oven temperature was initially 80 °C (held for 1 min) and was then increased at a rate of 30 °C/min up to 180 °C and held for 1 min, increased at a rate of 10 °C/min up to 220 °C and held for 1 min, and subsequently increased at a rate of 40 °C/min up to 270 °C and held for 1 min. The detector temperature was set at 280 °C. N_2_ (purity > 99.999%) was used as the carrier gas, and the flow rate was 1.5 mL/min.

### 3.4. Data Analysis

Expression of prochloraz residues was performed essentially as described previously [23]. The relationship between prochloraz residues and time is *C*_t_ = *C*_0_ × e^−kt^, where *C*_t_ (mg·kg^−1^) is the residue after time t, *C*_0_ (mg·kg^−1^) is the initial residue, e is a mathematical constant, and k is the dissipation rate constant (d^−1^). This equation is widely used to assess the level of pesticides in agriproducts [27,28].

## 4. Conclusions

In this study, the residue dynamics and risk assessment of prochloraz and 2,4,6-TCP in apples under different treatment concentrations were studied using a GC-ECD method. The derivatization percent of prochloraz to 2,4,6-TCP was stable and complete. Under the application of 2 °C 2.0 g/L, 2 °C 1.0 g/L, 20 °C 2.0 g/L, and 20 °C 1.0 g/L treatment, prochloraz residues in the whole fruit and apple peel samples showed gradual and continuous deterioration; the half-life for the degradation of prochloraz in the whole fruit was 69.3 d, 69.3 d, 63.0 d, and 57.8 d, respectively, and in the apple peel was 86.6 d, 77.0 d, 69.3 d, and 63.0 d, respectively; the concentration of prochloraz in the apple pulp increased gradually until a peak (0.72 mg·kg^−1^) was reached. 2,4,6-TCP residues in the whole fruit samples showed a gradual and continuous increase. The concentration of 2,4,6-TCP was below 0.1 mg·kg^−1^ in four treatment conditions, and 2,4,6-TCP was not detected (<LOD) in apple pulp. Finally, in 20 authentic apple samples on the Hefei market, the prochloraz concentration did not exceed the MRL of Chinese standards in apples (MRL = 2.0 mg·kg^−1^). However, it cannot be ignored that the MRL set by European Communities [29] and Japan Food Chemical Research Foundation [The Japan Food Chemical Research Foundation, http://www.m5.ws001.squarestart.ne.jp/foundation/agrdtl.php?a_inq=64900] for the sum of prochloraz and its metabolites in apples is 0.05 mg·kg^−1^, compared to the MRL of prochloraz (2.0 mg·kg^−1^) in apples that established by Chinese regulation [26], we know that the MRLs of the same hazardous substance vary greatly in different regions.

## Figures and Tables

**Table 1:** Derivatization percent of prochloraz to 2,4,6-TCP.

Concentration (mg·kg^−1^)	Derivatization Percent (%)	Average Percent (%)	RSD ^a^ (%)
0.1	94.9	101.0	4.2
0.2	106.7		
0.8	102.2		
1.0	100.2		

^a^ RSD: Relative standard deviations.

**Table 2:** Recovery studies of samples spiked with prochloraz and 2,4,6-TCP by GC-ECD.

Analyte	Sample	Spiked Level (mg·kg^−1^)	Recovery (%)	CV ^a^ (%)
Prochloraz	apple fruit	0.2	103.4	2.85
		1.0	109.2	3.49
		2.0	113.7	7.00
	apple pulp	0.2	114.4	1.55
		1.0	111.9	4.46
		2.0	82.9	5.71
	apple peel	0.25	93.9	8.61
		1.0	85.1	5.07
		5.0	87.4	3.52
2,4,6-TCP	apple fruit	0.02	85.0	5.19
		0.2	89.8	0.70
		2.0	87.8	0.65
	apple pulp	0.02	109.1	1.56
		0.2	84.7	2.14
		2.0	86.7	4.59

^a^ CV: coefficient of variation (*n* = 3).

**Table 3:** Statistical data of prochloraz dissipation in apple fruit and apple peel.

Sample	Treatment	Equation	T_1/2_ (d) ^a^	R^2 b^
Apple fruit	2 °C 2.0 g/L	C = 5.8001 × e^−0.010t^	69.3	0.9036
	2 °C 1.0 g/L	C = 3.9000 × e^−0.010t^	69.3	0.9505
	20 °C 2.0 g/L	C = 3.8852 × e^−0.011t^	63.0	0.9195
	20 °C 1.0 g/L	C = 3.3800 × e^−0.012t^	57.8	0.9739
Apple peel	2 °C 2.0 g/L	C = 20.953 × e^−0.008t^	86.6	0.7495
	2 °C 1.0 g/L	C = 18.970 × e^−0.009t^	77.0	0.8644
	20 °C 2.0 g/L	C = 18.184 × e^−0.010t^	69.3	0.9478
	20 °C 1.0 g/L	C = 13.123 × e^−0.011t^	63.0	0.8646

^a^ T_1/2_: The half-life; ^b^ R^2^: Correlation coefficient.

**Table 4:** Residues of prochloraz in authentic apple samples.

Number	Measured ± SD (mg·kg^−1^)	Number	Measured ± SD (mg·kg^−1^)	Number	Measured ± SD (mg·kg^−1^)
1	<LOD ^a^	8	<LOD	15	<LOD
2	<LOD	9	<LOD	16	0.86 ± 0.03
3	0.86 ± 0.01	10	0.49 ± 0.02	17	<LOD
4	<LOD	11	0.33 ± 0.02	18	<LOD
5	0.23 ± 0.01	12	0.65 ± 0.05	19	<LOD
6	0.63 ± 0.03	13	<LOD	20	<LOD
7	<LOD	14	<LOD		

^a^ LOD: <limited of detection.
